# Raman profiles of the stratum corneum define 3 filaggrin genotype–determined atopic dermatitis endophenotypes

**DOI:** 10.1016/j.jaci.2010.04.038

**Published:** 2010-09

**Authors:** Gráinne M. O'Regan, Patrick M.J.H. Kemperman, Aileen Sandilands, Huijia Chen, Linda E. Campbell, Karin Kroboth, Rosemarie Watson, Marion Rowland, Gerwin J. Puppels, W.H. Irwin McLean, Peter J. Caspers, Alan D. Irvine

**Affiliations:** a National Children's Research Centre, Our Lady's Children's Hospital, Dublin, Ireland; b Department of Paediatric Dermatology, Our Lady's Children's Hospital, Dublin, Ireland; c Department of Dermatology and Venereology, Erasmus MC, Rotterdam, The Netherlands; d Epithelial Genetics Group, Medical Sciences Institute, University of Dundee, Dundee, United Kingdom; e UCD School of Medicine and Medical Sciences, Dublin, Ireland; f Center for Optical Diagnostics and Therapy, Department of Dermatology and Venereology, Erasmus MC, Rotterdam, The Netherlands; g River Diagnostics BV, Rotterdam, The Netherlands; h Department of Medicine, Trinity College, Dublin, Ireland

**Keywords:** Atopic dermatitis, confocal Raman spectroscopy, eczema, filaggrin, hyperlinearity, natural moisturizing factor, transepidermal water loss, tyrosine, AD, Atopic dermatitis, AUC, Area under the curve, *FLG*, Filaggrin, HLP, Hyperlinear palms, NMF, Natural moisturizing factor, ROC, Receiver operating characteristic, SC, Stratum corneum, TEWL, Transepidermal water loss

## Abstract

**Background:**

Filaggrin (*FLG*) has a central role in the pathogenesis of atopic dermatitis (AD). *FLG* is a complex repetitive gene; highly population-specific mutations and multiple rare mutations make routine genotyping complex. Furthermore, the mechanistic pathways through which mutations in *FLG* predispose to AD are unclear.

**Objectives:**

We sought to determine whether specific Raman microspectroscopic natural moisturizing factor (NMF) signatures of the stratum corneum could be used as markers of *FLG* genotype in patients with moderate-to-severe AD.

**Methods:**

The composition and function of the stratum corneum in 132 well-characterized patients with moderate-to-severe AD were assessed by means of confocal Raman microspectroscopy and measurement of transepidermal water loss (TEWL). These parameters were compared with *FLG* genotype and clinical assessment.

**Results:**

Three subpopulations closely corresponding with *FLG* genotype were identified by using Raman spectroscopy. The Raman signature of NMF discriminated between *FLG*-associated AD and non–*FLG*-associated AD (area under the curve, 0.94; 95% CI, 0.91-0.99). In addition, within the subset of *FLG*-associated AD, NMF distinguished between patients with 1 versus 2 mutations. Five novel *FLG* mutations were found on rescreening outlying patients with Raman signatures suggestive of undetected mutations (R3418X, G1138X, S1040X, 10085delC, and L2933X). TEWL did not associate with *FLG* genotype subgroups.

**Conclusions:**

Raman spectroscopy permits rapid and highly accurate stratification of *FLG*-associated AD. *FLG* mutations do not influence TEWL within established moderate-to-severe AD.

*Discuss this article on the JACI Journal Club blog*: www.jaci-online.blogspot.com.

Atopic dermatitis (AD) is a complex and heterogeneous inflammatory skin disease driven and modified by immunologic, environmental, and genetic factors.^1,2^ The identification of filaggrin (*FLG*) null alleles in up to 50% of patients with moderate-to-severe AD implicates a fundamental role for barrier homeostasis in this disease.^3-6^ Although the mechanisms leading to AD in *FLG* mutation carriers are unclear, the deficiency of *FLG* likely facilitates permeability of biologically active allergens and microbial colonization that subsequently trigger inflammatory cascades.^7^ The recent identification of a murine model for *FLG* deficiency, with the detection of a homozygous frameshift mutation in the *Flg* gene in *flaky tail* mice, should accelerate our understanding of pathogenic mechanisms and therapeutic intervention points in patients with AD.^8^

Knowledge of the biochemical functions of filaggrin and its breakdown products indicate that a quantitative variation in gene or protein dosage might be relevant in determining phenotype.^9,10^ Filaggrin is initially produced as profilaggrin, a large, insoluble, heavily phosphorylated protein consisting of 10 to 12 tandem repeats of filaggrin units separated by short hydrophobic linker peptides.^11,12^ In the transitional layer profilaggrin is dephosphorylated and proteolytically processed into its functional filaggrin units, which bind to and collapse the keratin cytoskeleton and other intermediate filaments, acting as a scaffold for the subsequent reinforcement steps of the stratum corneum (SC).^13-15^ Subsequently, filaggrin is progressively degraded within the SC into a pool of hygroscopic amino acids, including pyrrolidone carboxylic acid, urocanic acid, and alanine. This composite mixture of amino acids and their derivatives, together with specific salts and sugars, form the natural moisturizing factor (NMF).^16,17^

NMF is highly hygroscopic and plays a central role in maintaining hydration of the SC and is additionally proposed to have a significant role in maintenance of the pH gradient of the skin, cutaneous antimicrobial defense, and regulation of key enzymatic events in the SC.^18,19^ Filaggrin thus has complex functions, with roles in establishing structural and chemical barrier function, hydration, and maintenance of epidermal homeostasis in the face of continuous transformation.^20^ Expression of filaggrin and subsequent breakdown of filaggrin into NMF is additionally determined based on properties of the microenvironment, including local pH, relative humidity, and protease activity.^17,21,22^*In vitro* evidence also indicates that filaggrin skin expression might be modulated by the atopic inflammatory response mediated by the cytokines IL-4 and IL-13.^23^ Genetically determined modifiers of protein dosage include the copy number of filaggrin repeat units, which vary in the population from 10 to 12 units and segregate by normal Mendelian genetic mechanisms.^11,24^ These genetic polymorphisms reflect tandem duplications of *FLG* repeats 8, 10, or both and might be an additional modifier of disease phenotype in heterozygotes who carry longer-sized variants on the unaffected allele.^25^ Our early data suggest that NMF levels correlate with *FLG*-null allele status and might therefore directly contribute to the dry skin phenotype seen in both patients with ichthyosis vulgaris and those with AD.^10^

Raman spectroscopy is capable of measuring *in vivo* information regarding the molecular composition of the skin, including quantitative analysis of amino acids and water content. It is based on the inelastic light scattering, or Raman scattering, of monochromatic light when the frequency of photons, usually from a laser source, changes on interaction with a sample, giving rise to characteristic Raman spectra and providing noninvasive real-time signatures of biological samples at a molecular level.

We sought to determine whether specific Raman NMF signatures of the SC could be used as markers of *FLG* genotype in patients with moderate-to-severe AD. We examined the association of NMF estimation with clinical evidence of hyperlinear palms (HLP), a clinical sign that has been shown to be associated with *FLG* mutations in previous studies. We also sought to examine the effects, within patients with moderate-to-severe AD, of *FLG* genotype on transepidermal water loss (TEWL) as a measure of an inside-out barrier defect.

## Methods

Following standard genetic practice, in this article *FLG*^−/−^ designates a patient homozygous for null alleles (ie, 2 null alleles), *FLG*^+/−^ designates a heterozygote null allele/wild-type (ie, 1 null allele), and *FLG*^+/+^ designates a homozygote wild-type (ie, 0 null alleles). A further abbreviation describes patients with AD with *FLG* mutations (*FLG*^+/−^ and *FLG*^−/−^) as AD*_FLG_* and those without *FLG* mutations (ie, *FLG*^+/+^) as AD*_NON-FLG_*.

One hundred thirty-five unrelated Irish children with a history of moderate-to-severe AD were recruited from dedicated tertiary referral AD clinics. Diagnosis was made by experienced pediatric dermatologists according to the United Kingdom diagnostic criteria.^26^ Exclusion criteria from the study were patients who had received systemic therapy, such as corticosteroids or immunosuppressants, in the preceding 3 months and patients whose ancestry was not exclusively Irish (4/4 grandparents). Detailed phenotypic data were collected. The Nottingham Eczema Severity Score^27^ was selected as an estimate of disease severity. Given previous publications and our own clinical experience of noting palmar hyperlinearity, this clinical sign was scored by using an investigator assessment of 0 (no hyperlinearity), 1 (mild/subtle hyperlinearity), and 2 (severe hyperlinearity).

### Genetic screening

All patients were screened for the 6 most prevalent *FLG* mutations in the Irish population (R501X, 2282del4, R2447X, S3247X, 3702delG, and Y2092X), as previously described.^28^ Full sequencing of *FLG* was performed as detailed previously.^28^ Based on screening for these 6 prevalent *FLG* mutations, 58.3% were carriers of 1 or more *FLG* mutations (15.1% *FLG*^−/−^, 43.2% *FLG*^+/−^, and 41.7% *FLG*^+/+^). An additional rare mutation was known to be present in 1 subject in a heterozygous state (R1474X). Additional screening was performed by means of complete sequencing of the *FLG* gene in selected subjects, as previously described.^29^ The entire collection was then rescreened for these 5 novel mutations found on complete sequencing.

### Biophysical analysis of the SC

Skin biophysical measurements were performed under standardized conditions (room temperature, 22 °C-25 °C; humidity levels, 30% to 35%). Before measurements, patients were acclimatized for a minimum of 10 minutes. All measurements were performed by one of 2 investigators (G.M.O. and P.M.J.H.K.). Topical therapies, including emollients, were withheld from the measurement sites for 48 hours preceding the study. TEWL was measured on nonlesional skin of the extensor forearm (Tewameter 300; Courage and Khazaka Electronic GmbH, Cologne, Germany).

NMF was measured in the SC of the thenar eminence by using confocal Raman microspectroscopy (model 3510 Skin Composition Analyzer; River Diagnostics, Rotterdam, The Netherlands). The principles of this method and the procedure have been described elsewhere.^30,31^ Depth profiles of Raman spectra were measured at 5-μm intervals from the skin surface at 30, 35, 40, 45 and 50 μm below the skin surface. An average of 8 profiles (totaling to 40 Raman spectra) from different areas of the thenar eminence were measured per patient. Raman spectra were recorded in the spectral region at 400 to 1,800 cm^−1^ with a 785-nm laser. Laser power on the skin was 25 mW. Levels of skin constituents relative to keratin were determined from the Raman spectra by means of classical least-squares fitting. Details of the method have been described elsewhere.^30,31^ Briefly, reference spectra of keratin, NMF, urocanic acid, lactate, urea, ceramide, and cholesterol were fitted to the individual Raman spectra from the skin. The combination of these reference spectra provides an adequate model for *in vivo* Raman spectra of normal human SC. A spectrum of reagent-grade L-tyrosine (Sigma-Aldrich, Zwijndrecht, The Netherlands) was added to this set of reference-fit spectra to enable determination of increased tyrosine levels. The resulting fit coefficients represent the relative proportions in which the skin constituents contribute to the total Raman skin spectrum. The reference spectrum of NMF had been constructed from the weighted sum of the spectra of its dominant constituents (pyrrolidone carboxylic acid, ornithine, serine, proline, glycine, histidine, and alanine). All concentrations of skin constituents relative to keratin were calculated from the recorded Raman spectra by using SkinTools 2.0 (River Diagnostics B.V., Rotterdam, The Netherlands); NMF levels derived from the individual Raman spectra were used to assess intrapatient variation in NMF. These NMF levels were then averaged to obtain the mean NMF level per patient. Of the 135 subjects who participated in the study, 3 patients were unable to fully cooperate with the Raman measurement, and their results were excluded because of insufficient spectra collection.

The study was conducted in accordance with the Declaration of Helsinki and was approved by the Research Ethics Committee of Our Lady's Children's Hospital, Dublin. Written informed consent was obtained from all patients or their parents.

### Statistical methods

Patients were characterized, *a priori*, into 3 genotypes (*FLG*^+/+^, *FLG*^+/−^, and *FLG*^−/−^), as described in the methods. The designation *FLG-associated AD (*AD*_FLG_*) includes those with 1 or 2 *FLG* mutations (*FLG*^+/−^ and *FLG*^−/−^), whereas non–*FLG*-associated AD (AD*_NON-FLG_)* are *FLG*^+/+^. The data were characterized as having either a normal or a skewed distribution. Positively skewed data were log transformed. Box and whiskers plots showing the median value with interquartile range (25th-75th box length) of variables were constructed for the 3 *FLG* genotypes (*FLG*^−/−^, *FLG*^+/−^, and *FLG*^+/+^). ANOVA was used to compare means among the 3 genotype subgroups and presented as means with SDs and 95% CIs. Homogeneity of variances was tested by using the Levine statistic. A *post hoc* Tukey analysis for multiple comparisons was performed to examine pairwise differences among the 3 genotype subgroups. Mean differences for each pairwise comparison was presented together with a 95% CI of the mean difference.

Nonparametric receiver operating characteristic (ROC) curves, which plot sensitivity against 1−specificity, were constructed to examine the utility of Raman spectroscopy to, in the first instance, differentiate between AD*_FLG_* and AD*_NON-FLG_.* We then sought to further determine whether, within the *FLG*-associated AD group, ROC curve analysis could determine an NMF cutoff point to predict homozygous or heterozygous subjects (*FLG*^−/−^ vs *FLG*^+/−^). Areas under the curve (AUCs) with 95% CIs are presented. The cutoff for maximal sensitivity and specificity was deduced from the graphic output. Sensitivity and specificity for our dataset, based on the ROC output, are also presented to provide a preliminary indication of the clinical utility of Raman spectroscopy as a novel technique to distinguish *FLG* genotypes in patients with AD. However, further evaluation of Raman spectroscopy will be required in different well-characterized populations of patients with AD and healthy subjects.

HLP was scored clinically as absent (0) mild (1), or severe (2). For statistical analysis, HLP was dichotomized to no evidence of hyperlinearity (clinical score 0) versus any hyperlinearity (clinical scores of 1 or 2). The κ statistic was used to measure agreement between clinical scores of HLP and an NMF cutoff of 1.07. Where the observed agreement between scores is better than the degree of agreement expected by chance alone, κ is scored from 0 to 1.0, with 1.0 representing perfect agreement. A κ value of less than 0.2 is poor, a score of 0.2 to 0.4 is fair, 0.4 to 0.6 is considered moderate agreement, 0.6 to 0.8 is considered good agreement, and greater than 0.8 is considered excellent agreement.^32^ Data were analyzed with SPSS software (version 15; SPSS, Inc, Chicago, Ill). Significance was set at the 5% level.

## Results

**Fig 1:**
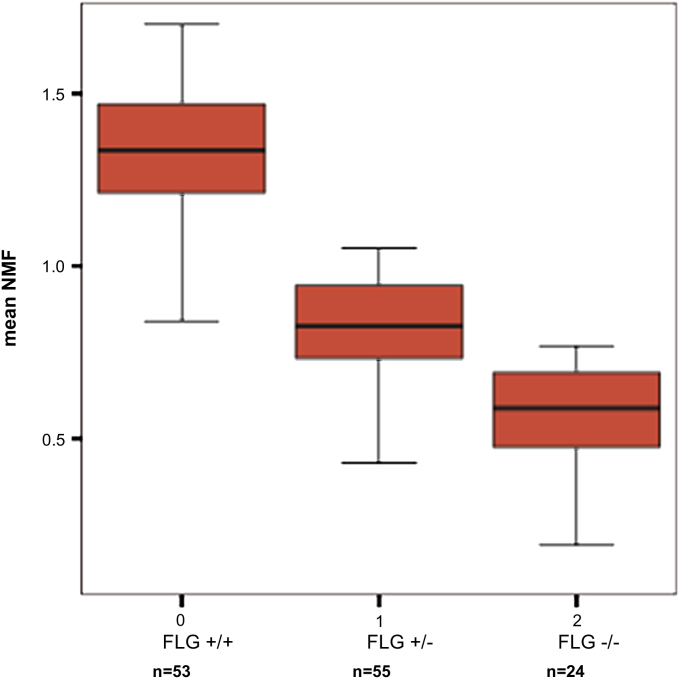
Box and whiskers plot of NMF by *FLG* genotypes (final genotype after full screening) showing the median *(midline)* and interquartile range corresponding to the length of the box.

Clinical characteristics and summary data of the study cohort are outlined in Table I. NMF levels assessed by means of Raman spectroscopy for subjects according to final *FLG* genotype (final genotype after full screening) are shown in Fig 1. In each pairwise comparison of the 3 genotypes, there is a statistically significant difference in NMF values (*P* < .001, ANOVA, Tukey post hoc analysis; Table II). IgE levels were not normally distributed. After log transformation of IgE data, there was no statistically significant difference in IgE values among the 3 *FLG* mutation subgroups (*P* < .48, Table I).

**Fig 2:**
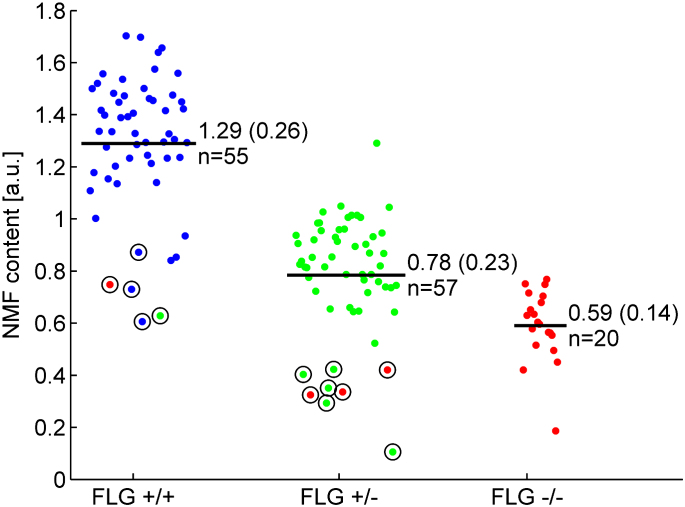
NMF cloud plot of values subcategorized after initial screening of 6 prevalent mutations. *Circles* indicate outliers who were rescreened for additional mutations. *Colors* indicate the final genotype after full screening. For each group, the number of patients and average NMF level (mean ± SD) are indicated in the figure. Group comparisons showing means and standard deviations by using ANOVA are presented in the text. *a.u.*, Arbitrary units.

**Fig 3:**
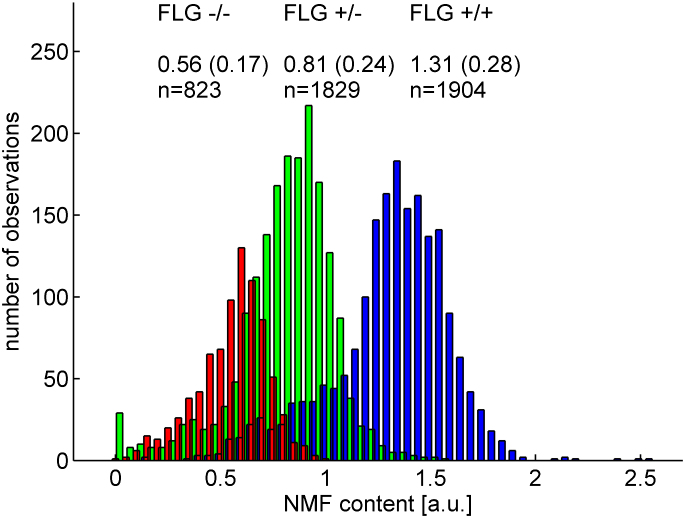
Histogram of all nonaveraged NMF values subcategorized according to corrected mutational status. *Blue bars* are *FLG*^+/+^ subjects, *green bars* are *FLG*^+/−^ subjects, and *red bars* are *FLG*^−/−^ subjects. For each group, the number of Raman measurements and the average NMF level (mean ± SD) are indicated in the figure. *FLG* genotype group sizes are as follows: *FLG*^−/−^, 24; *FLG*^+/−^, 55; *FLG*^+/+^, 53. *a.u.*, Arbitrary units.

Within the *FLG*^+/+^ and *FLG*^+/−^ genotype subgroups, 13 subjects had significantly outlying values with significantly lower NMF values compared with others who shared these genotypes (Fig 2). We hypothesized that these outlying values were suggestive of additional undetected *FLG* mutations. These 13 subjects were therefore fully sequenced for potential additional mutations. After full sequencing of these 13 subjects, 5 novel mutations (R3418X, G1138X, S1040X, 10085delC and L2933X) were detected. The entire collection was then rescreened to include 11 mutations (6 on initial screening plus these 5 additional mutations); however, no additional patients were found to harbor these novel rare mutations. NMF values by genotype after this additional screening are plotted in histogram format in Fig 3. Within *FLG* genotype subgroups, a range of values was seen; these values approximate to a normal distribution in each of the 3 genotype subgroups. The new mutations increased the percentage of patients with 1 or more *FLG* mutations in the collection to 59.8% (18.2% *FLG*^−/−^, 41.7% *FLG*^+/−^, and 40.2% *FLG*^+/+^; Table I).

### NMF values predict *FLG* genotype in patients with moderate-to-severe AD

**Fig 4:**
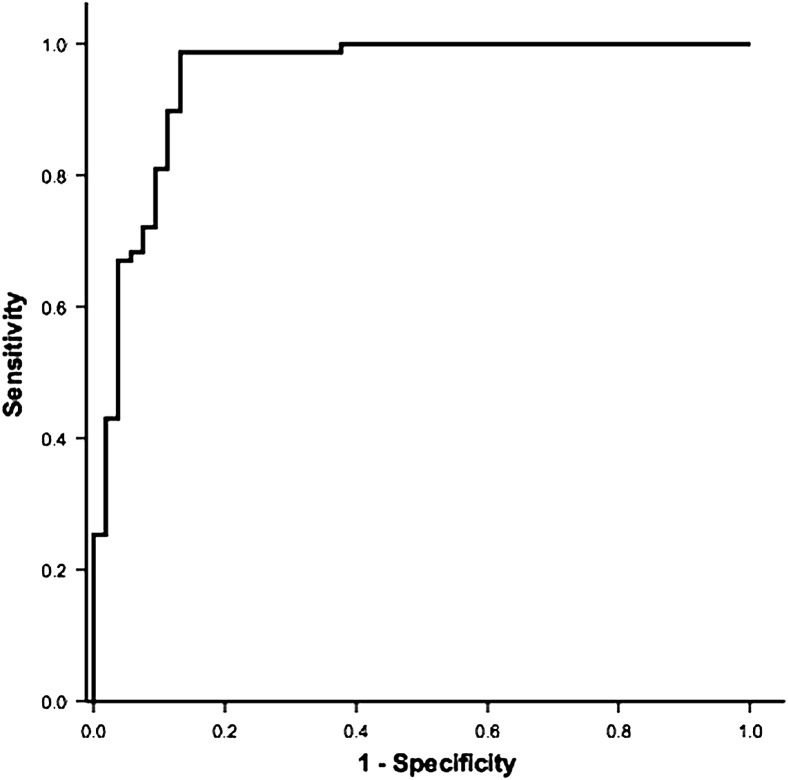
ROC curve for NMF AD*_FLG_* (genotype +/− and −/−) compared with AD*_NON-FLG_* (genotype +/+). The AUC is 0.95 (95% CI, 0.91-0.99). The optimal cutoff point for mean NMF to distinguish AD*_FLG_* from AD*_NON-FLG_* was 1.07 arbitrary units.

ROC curves were constructed to examine the discriminatory power of Raman-determined NMF. From a clinical decision tree, patients were initially grouped as AD*_FLG_* and AD*_NON-FLG_* (*FLG*^+/+^). The discriminatory power of Raman-determined NMF here was high, with an AUC on ROC analysis of 0.95 (95% CI, 0.91-0.99; Fig 4). The optimal cutoff value for NMF was 1.07, which would equate to a sensitivity of 98.73% and a specificity of 86.89% extrapolated from our ROC analysis. With the AD*_FLG_* group of children, NMF values also distinguished between *FLG*^+/−^ and *FLG*^−/−^ subjects, with an ROC AUC of 0.85 (95% CI, 0.77-0.93; see Fig E1 in this article's Online Repository at www.jacionline.org). This sensitivity and specificity is presented to suggest the probable utility of Raman spectroscopy as a novel diagnostic technique in patients with AD and is not presented as a test for the goodness of fit of the model.

### Tyrosine peaks on Raman microspectroscopy predict *FLG*^−/−^ genotype

**Fig 5:**
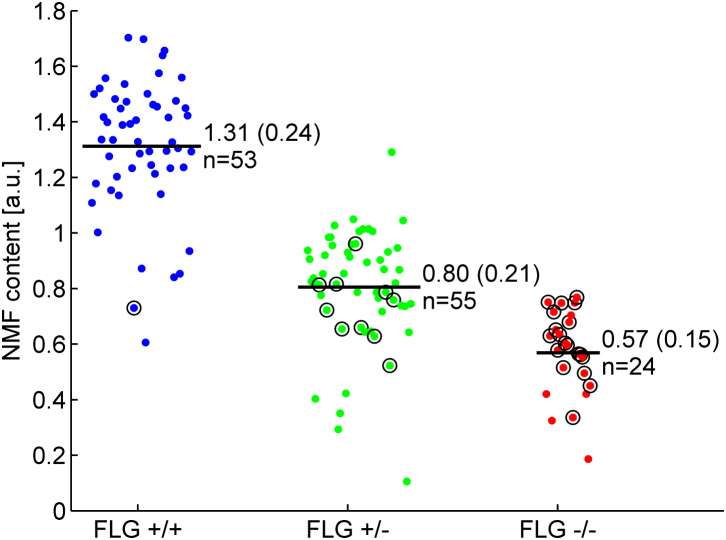
Cloud plot of NMF values categorized by genotype (final genotype after full screening). *Circles* indicate subjects with an increased tyrosine signal (*FLG*^+/+^, n = 1; *FLG*^+/−^, n = 10; *FLG*^−/−^, n = 19). For each group, the number of patients and average NMF level (mean ± SD) are indicated in the figure. *a.u.*, Arbitrary units.

The Raman spectra collected in the study revealed a distinct signal profile in a subset of patients subsequently identified by matching of *in vitro* Raman spectra of the amino acid tyrosine. Tyrosine levels 5 SDs greater than the normal tyrosine level measured in *FLG*^+/+^ subjects were identified as increased. Above this threshold, distinct tyrosine peaks could be visually identified in the corresponding Raman spectra. Tyrosine peaks were present in 19 (79%) of 24 of the patients who had 2 *FLG* mutations (Table I) and absent from all but 1 *FLG*^+/+^ subject, who also had an outlying low NMF level (Fig 5), suggesting an undetected genetic defect in *FLG*. The optimum NMF cutoff to correlate with tyrosine peaks was 0.831 (AUC, 0.81; 95% CI, 0.73-0.89), which is similar to the cutoff that distinguishes the *FLG*^−/−^ from the *FLG*^+/−^ populations (data not shown).

### TEWL in patients with moderate-to-severe AD does not discriminate between *FLG* genotype subgroups

There was no difference in mean TEWL levels among the 3 *FLG* genotype subgroups (Table I). TEWL did not distinguish *FLG* genotype status in patients with AD (TEWL AUC, 0.583; 95% CI, 0.48-0.699; ROC curve not shown).

### NMF values and palmar hyperlinearity scores

There was good agreement between clinical assessment of palmar hyperlinearity and an NMF cutoff of 1.07 (κ = 0.71). Thus palmar hyperlinearity might help distinguish patients with AD into those with 1 or more *FLG* mutations versus those with none. Clinical assessment is not as accurate as Raman spectroscopy in distinguishing the 3 *FLG* genotypes. The Nottingham Eczema Severity Score did not segregate with *FLG* genotype in this collection (data not shown), suggesting that the spectrum of AD severity within this moderate-to-severe collection is independent of *FLG* genotype.

## Discussion

A primary aim of this study was to investigate the utility of *in vivo* measurements of the SC to differentiate between *FLG* genotypes within patients with AD. Second, by making use of a very well-genotyped collection, we sought to determine whether *FLG* genotype influenced TEWL in patients with established moderate-to-severe AD. These results establish that the biochemical composition of the SC and palmar hyperlinearity associate with *FLG* mutation status, allowing rapid stratification of AD endotypes. Furthermore, we conclude that the degree of barrier defect, as measured by TEWL, in the clinically unaffected skin of patients with established AD is not influenced by *FLG* genotype.

The clinical utility of Raman-determined NMF is powerfully illustrated in the ROC curve analysis, which has an AUC of 0.95 (95% CI, 0.91-0.99) to discriminate between the AD*_FLG_* and AD*_NON-FLG_* groups. In addition, NMF can distinguish from heterozygous *FLG* mutations. In other words, this technique allows segregation of AD into *FLG* genotypes (+/+, +/−, and −/−) with such high specificity that it can accurately predict *FLG* genotype. Even in this relatively small patient population, mean NMF was statistically different between each of the 3 subpopulations (*post hoc* Tukey, Table II) Further validation of Raman-determined NMF is required in patients with well-characterized AD and healthy populations before this novel technique can be considered for clinical use.

The observed interindividual variation in NMF results within the mutation groups approximates to a normal distribution (Fig 3) that might be caused by environmental, genetic, or immunologic modifiers of *FLG* expression, including posttranslational processing. Homeostatic mechanisms or genetic mechanisms, such as the effects of copy number variation, small gene deletions, cytokine modulation of *FLG* expression, or upregulation of additional sources of NMF constituents, such as eccrine-derived lactate and glycerol, might account for differences in NMF levels within genotype groups. Modifying genes involved in filaggrin-processing pathways are likely to additionally contribute to this variation.^20^ The strong relationship between *FLG* mutation status and NMF implies that the role of other histidine-rich barrier proteins, such as the S100-fused proteins, including filaggrin-2 and hornerin, that are thought to have synergistic roles with filaggrin, including contribution to NMF levels,^33-35^ are unlikely to play a major role in the total NMF seen in AD patients.

The strong predictive value of a tyrosine signal in the SC of subjects with *FLG* mutations identifies a potential biomarker in the diagnosis of *FLG*-null subjects. Filaggrin lacks tyrosine; however, the linker segments and carboxyl-terminal domains of profilaggrin contain dense and highly conserved tyrosine-rich motifs.^11,12,36^ In normal SC only tyrosine incorporated in proteins, predominantly keratin, contributes to the overall Raman spectrum of skin; unbound tyrosine is usually not detectable. The consistent observation of the presence of tyrosine peaks in homozygous *FLG* mutation carriers might relate to altered biosynthetic pathways, such as limited proteolysis of profilaggrin or aberrant intermediate processing species, degraded through alternative pathways. The presence of tyrosine is independent of disease severity or mutation location.

Palmar and plantar hyperlinearity is a consistent clinical finding in the monogenic *FLG* disease ichthyosis vulgaris, and in keeping with a semidominant trait, intermediate hyperlinearity is thought to be associated with heterozygosity for *FLG* mutations; hyperlinearity has been associated as a significant finding in patients with *FLG*-related AD.^37-40^ Brown et al^41^ have recently shown significant association of palmar hyperlinearity with *FLG*-null mutations in a population-based cohort.^41^ Our finding that *FLG* status and NMF segregate with palmar hyperlinearity in patients with moderate-to-severe AD further validates the importance of this clinical sign in AD phenotypes.

Evidence from both molecular genetics and functional analyses strongly supports a skin barrier defect as a feature of both lesional and nonlesional skin in patients with AD.^42-44^ Increased mean baseline TEWL levels, reflecting a dysfunction of the inside-out epidermal permeability barrier in the clinically uninvolved skin of patients with AD, have been shown in many previous studies.^45-47^ The concept of an inherent skin barrier defect in both lesional and nonlesional skin from patients with is further supported by other methodologies, including recent data by Jakasa et al^43^ showing enhanced uptake of entire series of polyethylene glycols covering molecular weights in the range 150 to 590 daltons in nonlesional skin of patients with AD. Hata et al, using a photoacoustic spectroscopic system, showed enhanced penetration of both lipophilic and hydrophilic dye through clinically normal skin of patients with AD compared with that seen in control subjects.^42-44^

Previous investigators have reported contrasting findings on the influence of *FLG* mutation status on the inside-out barrier, as measured by TEWL in patients with AD; however, these studies were restricted by small sample size and possibly confounded by the technical limitations of disclosure of full *FLG* polymorphism status in the populations studied.^10,48^ A French study including a small number of *FLG* mutation carriers suggested that TEWL in patients with AD was not influenced by *FLG* status.^49^ Here, in our much larger collection of comprehensively genotyped patients with moderate-to-severe AD, TEWL again showed no discernable difference between *FLG* genotype subpopulations. These results suggest that increased TEWL in patients with moderate-to-severe AD is independent of *FLG* status and is a common end point in patients with moderate-to-severe AD. Thus increased TEWL might result from the systemic effects of the inflammatory process, even at nonclinically inflamed sites. Interestingly, flaky tail *(ft)* mice show a moderate increase in baseline TEWL, a deviation that increases dramatically after allergen priming and the subsequent secondary inflammatory response.^8^ In these mice, although *FLG* deficiency might of itself lead to an inside-out barrier defect barely measureable by increased TEWL, the subsequent marked increase in TEWL appears to be driven by the secondary immunologic response.

Within this collection, no effect was seen for *FLG* and disease severity or total serum IgE level; however, this case series is not the ideal collection to examine this because all cases are moderate to severe and therefore the full range of severity is not represented. Further population-based and sufficiently powered studies should clarify this potential genotype-phenotype correlation.

Within patients with AD, Raman signatures of NMF level and tyrosine predict *FLG* mutation status, overcoming the need for technically demanding genotyping, particularly in populations in which the *FLG* mutation architecture has not been comprehensively elucidated. This work additionally further informs mechanistic pathways in AD, demonstrating that TEWL in patients with moderate-to-severe AD is an outcome that is independent of *FLG* mutation status and pointing to a complex systemic interplay of environmental, immunologic, and functional pathways in the genesis of epithelial barrier disruption in patients with AD.

**Key messages**

• Raman spectroscopy, specifically NMF analysis, permits rapid and accurate stratification of FLG-associated versus non–FLG-associated AD and might be useful as a predictive test for FLG mutations.
• There is good agreement between FLG mutation status, NMF, and clinical palmar hyperlinearity scoring in patients with moderate-to-severe AD. Palmar hyperlinearity might help to define a clinical and genetic phenotype of AD.
• Within patients with moderate-to-severe AD, TEWL levels do not segregate with FLG genotype, suggesting that FLG mutations are neither necessary nor sufficient to fully explain this inside-out barrier defect in established AD.

## Figures and Tables

**Figure:**
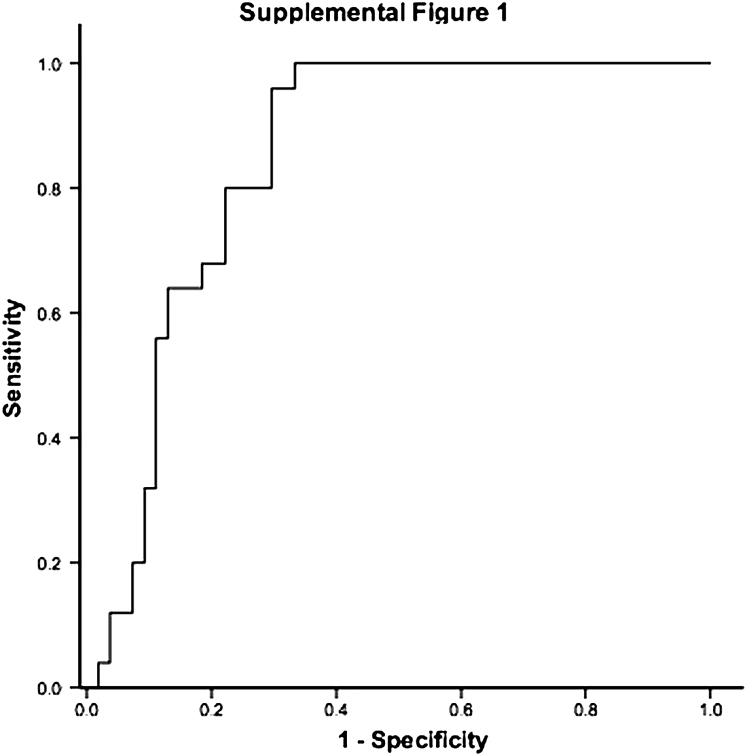
ROC curve for NMF values to distinguish *FLG*^+/−^ subjects from *FLG*^−/−^ subjects in the AD*_FLG_* group. AUC is 0.85 (95% CI, 0.76-0.93), with a sensitivity of 96% and a specificity of 66.77% at a cutoff of 0.767 arbitrary units.

**Table I:** Cohort characteristics according to final genotype

Final *FLG* genotype∗†	All mutations combined, no. (%)	Age (y), mean (SD)	Male sex, no. (%)	NESS,‡ mean (SD)	Palmar hyperlinearity score‡	TEWL (g/m^2^/h), mean (SD)	NMF (AU), mean (SD)	Tyrosine peaks, no. (%)	Log IgE# mean (SD)
Screened§	n = 132	n = 132	n = 132	n = 132	n = 131	n = 102	n = 132	n = 132	n = 129
+/+	53 (40.15)	8.43 (3.76)	32 (60.3)	12.40 (2.39)	0: 36	15.54 (9.34), n = 41	1.31 (0.24)	1 (1.89)	6.89 (2.29)
					1: 10				
					2: 7				
					n = 53				
+/−	55 (41.66)	8.2 (4.12)	32 (58.2)	11.28 (2.80)	0: 8	15.59 (6.72), n = 44	0.80 (0.21)	10 (18.18)	6.56 (1.78)
					1: 23				
					2: 23				
					n = 54				
−/−	24 (18.18)	8.83 (4.16)	15 (62.5)	12.13 (2.52)	0: 0	17.35 (7.37), n = 17	0.57 (0.15)	19 (79.16)	7.11 (1.50)
					1: 1				
					2: 23				
					n=24				
*P* value	−	.84‖	.97¶	.07‖	−	.70‖	<.0001‖	<.0001¶	.48‖

*AU*, Arbitrary units.

∗ Final *FLG* status after rescreening cohort for all mutations (*FLG* status before detection of novel mutations: *FLG*^+/+^, 41.7%; *FLG*^+/−^, 43.2%; and *FLG*^−/−^, 15.1%).

† *+/+*, No *FLG* mutation; *+/−*, 1 *FLG* mutation; *−/−*, 2 *FLG* mutations.

‡ *NESS*, Nottingham Eczema Severity Score. Palmar hyperlinearity score: 0, no hyperlinearity; 1, intermediate; 2, marked.

§ Hyperlinearity scoring and TEWL data are not available for 1 and 30 subjects, respectively.

‖ Comparison of means among the 3 groups of *FLG* mutations using ANOVA.

¶ Comparison of proportions using χ^2^ test for comparison of a 2 × 3 contingency table.

# IgE data were positively skewed. The mean of the log-transformed data is presented.

**Table II:** ANOVA showing a statistically significant difference in NMF among the 3 *FLG* genotype subgroups together with 95% CIs

Genotype	No.	Mean NMF	95% CI	Comparison genotype	Mean difference	95% CI mean difference	*P* value∗
*FLG*^+/+^	53	1.31 ± 0.24	1.24 to 1.37	*FLG*^+/−^	0.51	0.40 to 0.60	<.001
				*FLG*^−/−^	0.74	0.62 to 0.87	<.001
*FLG*^+/−^	55	0.80 ± 0.21	0.74 to 0.86	*FLG*^+/+^	−0.51	−0.60- to −0.41	<.001
				*FLG*^−/−^	0.24	0.11 to 0.36	<.001
*FLG*^−/−^	24	0.57 ± 0.15	0.50 to 0.63	*FLG*^+/+^	−0.74	−8.86 to −0.61	<.001
				*FLG*^+/−^	−0.24	−0.36 to −0.11	<.001

A *post hoc* analysis with Tukey correction shows a statistically significant difference in mean NMF between each pairwise comparison for the 3 *FLG* genotype subgroups.

∗ Tukey multiple comparisons.
